# *Flavobacterium okayamense* sp. nov. isolated from surface seawater

**DOI:** 10.1007/s00203-023-03682-x

**Published:** 2023-09-29

**Authors:** Kei Kitahara, Basilua Andre Muzembo, Sho Morohoshi, Tadao Kunihiro, Nozomi Tazato, Ayumu Ohno, Kazuma Uesaka, Makoto Taniguchi, Shin-ichi Miyoshi

**Affiliations:** 1 Graduate School of Medicine, Dentistry and Pharmaceutical Sciences, Okayama University, Okayama, Japan; 2 TechnoSuruga Laboratory Co., Ltd, Nagasaki, Shizuoka Japan; 3 Graduate School of Bioagricultural Sciences, Nagoya University, Nagoya, Japan; 4 Oral Microbiome Center, Taniguchi Dental Clinic, Takamatsu, Japan

**Keywords:** *Bacteroidota*, *Flavobacterium*, New taxa, Sea water

## Abstract

Strain KK2020170^T^, a Gram-stain negative, yellow colony-forming bacterium, was isolated from surface seawater sampled in Kojima Bay, Okayama, Japan. Phylogenetic analysis based on the 16S rRNA gene revealed that strain KK2020170^T^ belongs to the genus *Flavobacterium*, with *Flavobacterium haoranii* LQY-7^T^ (98.1% similarity) being its closest relative, followed by *Flavobacterium sediminis* MEBiC07310^T^ (96.9%) and *Flavobacterium urocaniciphilum* YIT 12746^T^ (96.0%). Whole-genome shotgun sequencing showed that strain KK2020170^T^, when paralleled with *F. haoranii* LQY-7^ T^, had 81.3% average nucleotide identity, and 24.6% in silico DNA–DNA hybridization values, respectively. The DNA G + C content of strain KK2020170^T^ was 31.1 mol%. The most abundant fatty acids (> 10%) of strain KK2020170^T^ were iso-C15: 0, iso-C17: 0 3-OH and iso-C15: 1 G. The dominant respiratory quinone of the strain was menaquinone MK-6. Based on the phylogenetic and phenotypic analysis results, we propose that strain KK2020170^T^ represents a novel species, for which the name *Flavobacterium okayamense* sp. nov. has been proposed. The type strain is KK2020170^T^ (= ATCC TSD-280^ T^ = NBRC 115344^ T^).

**Supplementary Information:**

The online version contains supplementary material available at 10.1007/s00203-023-03682-x.

## Introduction

The genus *Flavobacterium*, a member of the family *Flavobacteriaceae* within the phylum *Bacteroidota*, was proposed by Bergey et al. (Bergey et al. 1923; Zhang et al. 2019) and subsequently revised by Bernardet et al. (1996). This genus contains at least 314 species with legitimately published names at the time of writing (https://lpsn.dsmz.de/, April 2023). *Flavobacterium* species have been recognized in a wide variety of biotopes, including soil (Máté et al. 2022; Hou et al. 2022), river water (Watanabe et al. 2022), plants (Seo et al. 2022), seawater (Sun et al. 2022), sites in Antarctica (Králová et al. 2021), and chinstrap penguins (Irgang et al. 2023). Physiologically, cells of the genus *Flavobacterium* are typically Gram-stain-negative, rod-shaped, aerobic, and heterotrophic, and they yield yellow or orange colonies (McBride 2014). This study described the isolation of strain KK2020170^T^ from surface seawater in Kojima Bay, Okayama, Japan and examined whether this strain represents a new species under the genus *Flavobacterium*.

## Materials and methods

### Isolation and cultivation

Strain KK2020170^T^ was isolated from surface seawater in Kojima Bay (34° 6048′ N 133° 9856′ E) in Okayama, Japan via a traditional dilution-plate method on trypticase soy agar (TSA) (BBL, Becton Dickinson, Franklin Lakes, USA). Sampling was performed in late October 2020. The sampled seawater had a temperature of 22 °C, NaCl concentration of 1.15% (w/w), and pH of 7.79. A yellow colony on the TSA, which had been incubated for 72 h at 30 °C, was picked up and single-colony isolation processes were repeated three times to establish a pure culture. The isolated strain was routinely grown at 30 °C on TSA or in trypticase soy broth (TSB) (BBL, Becton Dickinson, Franklin Lakes, USA). To preserve the strain, an overnight TSB culture was mixed well with 20% (final v/v) glycerol and preserved at  −80 °C as a glycerol stock.

### Phylogeny

To examine the 16S rRNA gene, genomic DNA was isolated from the cell pellet of a 1-ml overnight liquid culture of strain KK2020170^T^ using a QIAamp DNA Mini Kit (Qiagen, Hilden, Germany) per the manufacturer’s instructions. The 16S rRNA gene was directly PCR-amplified from the template (genomic DNA) using a universal primer set for bacteria (27F and 1492R) (Lane 1991; Kitahara et al. 2012). The resulting PCR product, which was well separated as a single band (approximately 1500 bp) by agarose-gel electrophoresis, was excised from the gel and purified using a QIAquick Gel Extraction Kit (Qiagen, Hilden, Germany). The amplicon was directly sequenced using an ABI 3130XL automatic sequencer (Applied Biosystems, Waltham, USA) according to the supplier’s procedure. A similarity search of the sequenced 16S rRNA gene was performed using international nucleotide sequence databases (DDBJ/ENA/GenBank). Phylogenetic trees were built by the neighbor-joining (NJ) and maximum-likelihood (ML) algorithms (Felsenstein 1981; Fitch 1971; Saitou, and Nei 1987) using MEGA v.7.0 (Kumar et al. 2016). Each topology of the two phylogenetic trees was validated by 1000 random bootstrap replicates.

### Genome features

To obtain further DNA sequence information to strengthen the phylogenetic position of strain KK2020170^T^, whole-genome shotgun sequencing was performed using a MiSeq instrument (Illumina, San Diego, USA) and GridION X5 system (Oxford Nanopore Technologies, Oxford, UK). Although MiSeq can read short DNA fragments with high accuracy, GridION can read longer (thus structurally collect) DNA fragments. Combining the information of both techniques is effective for determining the complete genome of a bacterium in terms of ensuring both sequence accuracy and high structural integrity of the DNA (Miyazaki et al. 2020; Yu et al. 2019). The sequencing and data analysis methods used in this study are concordant to the specified basic standards for genome data for prokaryotic taxonomy (Chun et al. 2018).

### Physiology and chemotaxonomy

The physiological and chemotaxonomic properties of strain KK2020170^T^ were examined with reference to the minimal requirements for describing new species in *Flavobacterium* (Bernardet et al. 2002; Jung et al. 2017; Yang et al. 2016). Other experiments conducted in studies describing the closest relatives of strain KK2020170^T^ (*F. haoranii* LQY-7^ T^ (Zhang et al. 2010), *F. sediminis* MEBjC07310^T^ [Bae et al. 2018), *F. indicum* GPTSA100-9^ T^ (Saha and Chakrabarti 2006) and *F. urocaniciphilum* YIT 12746^ T^ (Fujii et al. 2014)] were also performed. Unless otherwise noted, the isolate (strain KK2020170^T^) was routinely grown on TSA or in TSB at 30 °C for morphological and biochemical characterization.

To observe the morphology of strain KK2020170^T^ cells, cells were cultured on TSA at 30 °C for 72 h. Harvested cells were Gram-stained using Favor G Nissui (Nissui Pharmaceutical, Tokyo, Japan) per the supplier’s protocols, and they were visualized under a phase-contrast microscope (BX50F4; Olympus, Tokyo, Japan) at × 1000 magnification. Colony morphology was observed after incubating cells at 30 °C for 72 h. Growth phenotypes at wide ranges of temperatures (4, 10, 15, 20, 25, 30, 35, 40, and 45 °C) were tested on TSA for 72 h. Salt sensitivity was tested in TSB supplemented with different final concentrations of NaCl (0%–8% w/v, increased in 1% increments). The pH sensitivity of strain KK2020170^T^ was tested in TSB with a series of different pH from 4.5 to 10.0 in 0.5-unit increments [pH was fine-tuned using 10 mM MES (pH 4–6) or 10 mM Tris (pH 7–10) buffers]. The existence of flexirubin-type pigments in colonies was evaluated using previously reported methods (Bernardet et al. 2002). Catalase and oxidase activities were evaluated using 3% (v/v) H_2_O_2_ and 1% (w/v) tetramethyl-p-phenylenediamine, respectively, using previously described methods (Smibert et al. 1994). Growth ability was assessed on Marine Broth 2216 agar (Becton Dickinson, Franklin Lakes, USA), nutrient agar (Oxoid, Basingstoke, UK), and MacConkey agar (Nissui Pharmaceutical, Tokyo, Japan). Casein and starch hydrolysis was examined for 7 days at 30 °C according to standard protocols (Smibert, and NR. 1994; Cowan 1965). Acid production from carbohydrates, enzyme activities, and other biochemical features of strain KK2020170^T^ were evaluated using API 20NE and API ZYM (BioMérieux, Marcy-l'Étoile, France), both of which are widely used for phenotype-based bacterial characterization or identification purposes, following the manufacturer’s protocols. Susceptibility to the following antibiotics was tested by the disc diffusion procedure (µg/disc) on TSA for 24 h at 30 °C using Sensi-Disc (Becton Dickinson, Franklin Lakes, USA): amoxicillin (25), ampicillin (10), bacitracin (10), carbenicillin (100), chloramphenicol (30), erythromycin (15), gentamicin (10), kanamycin (30), lincomycin (2), rifampicin (5), penicillin G (10), polymyxin B (300), tetracycline (30), spectinomycin (100), streptomycin (10), and vancomycin (30). The anaerobic growth test was performed by incubating a TSA plate at 37 °C for 72 h, and oxygen was absorbed using an Anaero Pack gas system (Anaero Pack disposable, Mitsubishi Gas Chemical, Tokyo, Japan).

To compare fatty acid compositions, reference strains (*F. haoranii* LQY-7^ T^ and *F. sediminis* MEBiC07310^T^) were procured from the Japan Collection of Microorganisms and Leibniz Institute DSMZ–German Collection of Microorganisms and Cell Cultures GmbH, respectively.

The fatty acids of strain KK2020170^T^ and the reference strains (LQY-7^ T^ and MEBiC07310^T^) were prepared per the standard MIDI protocol (Sasser 1990) by culturing on TSA at 28 °C for 24 h. The fatty acid methyl esters were examined by gas chromatography (7890A, Agilent Technologies, Santa Clara, USA) and identified using the TSBA6 database of the Microbial Identification System (Sherlock, v. 6.0). Isoprenoid quinones were extracted using a method described by Bligh and Dyer (Bligh and Dyer 1959; Tamaoka et al. 1983) and analyzed using an ACQUITY UPLC H-Class system with a PDA detector (Waters, Milford, USA) using a reversed-phase BEH C18, 2.1 (I.D.) × 150 mm, 1.7 μm column (Waters). The polar lipids of strain KK2020170^T^ grown on TSA for 24 h at 28 °C were extracted and tracked by two-dimensional thin-layer chromatography as previously described (Minnikin et al. 1979).

## Results and discussion

### Phylogenetic analysis

**Fig. 1:**
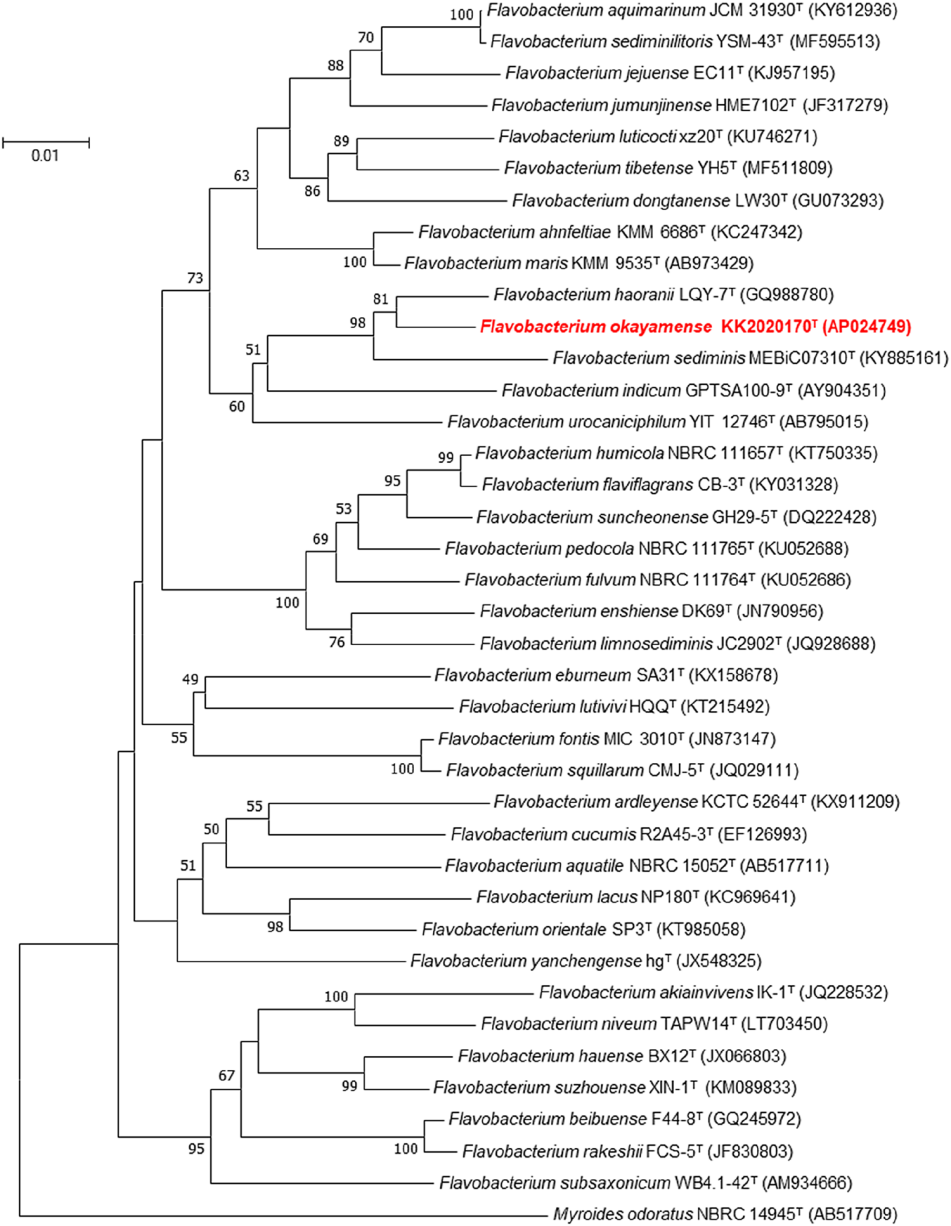
Neighbor-joining tree depicting the evolutionary relationships of *F. okayamense* strain KK2020170^T^ and 37 adjacent type strains in the genus *Flavobacterium* based on 16S rRNA gene sequences. Bootstrap values (given as percentages of 1000 replicates) with > 50% values are displayed. *Flavobacterium aquatile* is the type species in the genus *Flavobacterium*. *Myroides odoratus* NBRC 14945^ T^ (AB517709) was employed as an outgroup. The bar represents a Knuc distance of 0.01

Sequence similarity calculations of 16S rRNA gene (with 1439 unambiguously aligned base pairs) suggested that strain KK2020170^T^ was most closely related to *F. haoranii* LQY-7^ T^ (98.1% similarity) (Zhang et al. 2010), followed by *F. sediminis* MEBiC07310^T^ (96.9% similarity) (Bae et al. 2018) and *F. urocaniciphilum* YIT 12746^ T^ (96.0%) (Fujii et al. 2014). All other type strains had lower than 96.0% similarity. The NJ tree constructed from 16S rRNA gene sequences revealed that, strain KK2020170^T^ clearly belongs to the genus *Flavobacterium*, surrounded by legitimately named *Flavobacterium* members (Fig. 1). The same phylogenetic position of strain KK2020170^T^ was reproducibly observed in the ML-reconstructed phylogenetic tree (Fig. S1, available in the online supplementary materials). In general, it is empirically known that when a bacterial strain’s 16S rRNA gene sequence has lower than 98.7% similarity to the closest type strain, the strain can possibly be classified as a novel species (Chun et al. 2018). Our 16S rRNA gene-based data suggested that it is possible that the strain has a unique phylogenetic position at species level within the genus *Flavobacterium*.

### Whole-genome sequence analysis

The full genome of strain KK2020170^T^ was identified as a single-circular chromosome of 2,781,077 bp containing 2574 protein-coding genes, 9 rRNAs, and 51 tRNA genes (Table S1). The G + C content of the genome was 31.1 mol%, which was within the reported range for *Flavobacterium* strains (30 mol%–41 mol%) (Bernardet et al. 1923). To distinguish strain KK2020170^T^ from *F. haoranii* LQY-7^ T^ (ASM936305v1) and *F. sediminis* MEBiC07310^T^ (ASM314838v1), two independent in silico comparative approaches were applied. First, pyani v.0.2.10 was used to compute the average nucleotide identity (ANI) (Pritchard et al. 2016), and the ANI between strain KK2020170^T^ and *F. haoranii* LQY-7^ T^ was 81.3%, versus 75.8% between strain KK2020170^T^ and *F. sediminis* MEBiC07310^T^. When a bacterial strain, as a comparison to the closest type strain, has an ANI lower than the standard cutoff (95–96%), the strain has a high possibility of being classified as a new species. Next, in silico DNA–DNA hybridization (dDDH) using Genome-to-Genome Distance Calculator v.2.1 (http://ggdc.dsmz.de/) (Meier-Kolthoff et al. 2013) was applied. The mean genome-to-genome distance (in silico DDH) between strain KK2020170^T^ and *F. haoranii* LQY-7^ T^ was calculated as 24.6%. This value was 20.0% when strain KK2020170^T^ and *F. sediminis* MEBiC07310^T^ were compared. Both values were significantly below the conventional threshold (70%), which is used to distinguish two bacterial strains from each other at the species level. These genomic study results strongly suggest that strain KK2020170^T^ is a new species in *Flavobacterium*.

### Physiology and chemotaxonomy

Cells of strain KK2020170^T^ were Gram-stain-negative rods (0.5–0.6 × 1.0–9.5 μm, Fig. S2A). Gliding motility was not observed. Strain KK2020170^T^ colonies grown on TSA plate were yellow, circular, and smooth with low convex (Fig. S2B). In addition to aerobic growth, weak anaerobic growth was observed. Flexirubin-type pigments were not detected. Cells were susceptible to amoxicillin, ampicillin, carbenicillin, chloramphenicol, erythromycin, lincomycin, penicillin G, polymyxin B, rifampicin, spectinomycin, tetracycline, and vancomycin but resistant to bacitracin, gentamicin, kanamycin, and streptomycin. The physiological and biochemical properties used to differentiate strain KK2020170^T^ and closely connected *Flavobacterium* species are summarized in Table 1. Briefly, strain KK2020170^T^; unlike *F. haoranii* LQY-7^ T^, does not hydrolyze aesculin, gelatin, and starch. Conversely, strain KK2020170^T^, unlike *F. haoranii* LQY-7^ T^, hydrolyzes casein. Urease and α-chymotrypsin activities were observed for *F. haoranii* LQY-7^ T^ but not for strain KK2020170^T^.

**Table 1:** Differential phenotypic attributes of* F. okayamense* strain KK2020170^T^ and closely related *Flavobacterium* type strains

	1	2	3	4	5
Cell size (µm)	0.5–0.6 × 1.0–9.5	0.3–0.7 × 1.3–2.0	0.3–0.7 × 1–3	0.5–1.0 × 1.5–2.5	0.3–0.7 × 1–2.5
Growth					
Temperature (°C)	15–40 (30)	15–37 (30)	17–43 (32)	15–43 (37)	10–37 (30)
pH	6.5–8.5 (7)	6–8.5 (7)	5–8 (7)	5–11 (7.4–8)	6.5–8.5 (7)
NaCl concentration (%, w/v)	1–4 (1)	0–5 (1)	0–3 (1)	0–2	0–0.5 (0)
Growth on nutrient agar	+	+	−	W	+
Anaerobic growth	W	NR	−	−	+
Catalase	W	+	+	W	+
Hydrolysis of					
Aesculin	−	+	+	−	−
Casein	+	−	−	+	−
Gelatin	−	+	−	+	+
Starch	−	+	+	W	−
Tyrosine	−	−	−	−	+
Acid production from					
d-Glucose	−	−	−	+	−
Enzyme activity (API ZYM, API 20NE)					
Urease	−	+	–	NR	–
α-Chymotrypsin	−	+	+	NR	+
β-Glucuronidase	−	−	+	NR	−
DNA G + C content (mol%)	31.1	34	35.2	31	30.9

1, *F. okayamense* strain KK2020170^T^ (this study); 2, *F. haoranii* LQY-7^ T^ (Zhang et al. 2010); 3, *F. sediminis* MEBjC07310^T^ (Bae et al. 2018); 4, *F. indicum* GPTSA100-9^ T^ (Saha and Chakrabarti 2006); 5, *F. urocaniciphilum* YIT 12746^ T^ (Fujii et al. 2014). Ranges (minimal and maximal values) and the optimal values (in parentheses) are shown for temperature, pH and NaCl concentration

*+* Positive, *−*  negative, *W* weakly positive, *NR* not reported

The major fatty acids (> 10%) of strain KK2020170^T^ were iso-C15:0 (53.6%), iso-C15:1 G (12.9%), and iso-C17:03-OH (12.2%). In Table 2, the cellular fatty acid profile of strain KK2020170^T^ is paralleled with those of *F. haoranii* LQY-7^ T^, *F. sediminis* MEBiC07310^T^, *F. indicum* GPTSA 100-9^ T^, and *F. urocaniciphilum* YIT 12746^ T^. The fatty acid profile of strain KK2020170^T^ resembled that of *F. haoranii* LQY-7^ T^, in which the major detected components were iso-C15:0, iso-C15:1 G, and iso-C17:0 3-OH (Table 2). The fatty acid composition of strain KK2020170^T^ was distinguishable from those of *F. haoranii* LQY-7^ T^ and *F. sediminis* MEBiC07310^T^ in that iso-C14:0 and C17:1ω6c were not detected in strain KK2020170^T^. Menaquinone 6 (MK-6) was detected as the dominant (99.9%) respiratory quinone in strain KK2020170^T^, similar to the findings in other members of the family *Flavobacteriaceae*. Strain KK2020170^T^ exhibited a complex polar lipid profile consisting of one phosphatidylethanolamine as the dominant element, three aminolipids, one lyso-phosphatidyl-ethanolamine, and four unidentified lipids (Fig. S3).

**Table 2:** Cellular fatty acid proportions (%) of *F. okayamense* strain KK2020170^T^ and closely related *Flavobacterium* type strains

	1	2	3	4	5
Saturated					
C15:0	−	−	−	NR	**12.0**
C16:0	−	−	−	NR	3.4
Saturated branched-chain					
iso-C13:0	2.3	TR	TR	NR	TR
iso-C14:0	−	TR	−	NR	2.0
iso-C15:0	**53.6**	**44.2**	**34.4**	**18.5**	**21.5**
iso-C15:1 G	**12.9**	**17.0**	**11.74**	**18.0**	**11.9**
iso-C16:0	TR	TR	TR	5.1	**13.3**
iso-C16:1 G	−	−	−	−	2.7
iso-C16:1 H	−	TR	−	NR	−
anteiso-C15:0	1.1	2.8	TR	NR	TR
Unsaturated branched-chain					
C15:1ω6*c*	TR	TR	TR	NR	1.2
C17:1ω6*c*	−	TR	TR	NR	NR
Hydroxy					
C15:0 3-OH	−	−	−	NR	1.0
C16:0 3-OH	TR	TR	TR	NR	2.7
iso-C14:0 3-OH	TR	TR	TR	NR	1.1
iso-C15:0 3-OH	5.4	6.6	5.6	5.0	6.0
iso-C16:0 3-OH	TR	1.0	TR	4.5	5.6
iso-C17:0 3-OH	**12.2**	**15.5**	**17.2**	9.0	6.2
Summed features					
3 (C16:1ω7c and/or iso-C15:0 2-OH)	TR	TR	**16.4**	**16.6**	2.1
4 (C17:1 ISO I and C17:1 ANTEISO B)	TR	1.03	2.8	NR	NR
9 (C17:1 ISO ω9c and C16:0 10-methyl)	**10.0**	6.6	7.3	NR	NR

1, *F. okayamense* strain KK2020-76^ T^ (current study); 2, *F. haoranii* LQY-7^ T^ (this study); 3, *F. sediminis* MEBjC07310^T^ (this study); 4, *F. indicum* GPTSA 100-9^ T^ (Saha and Chakrabarti, 2006); 5. *F. urocaniciphilum* YIT 12746^ T^ (Fujii et al. 2014). Bold values > 10%; TR, < 1%; − , not detected, *NR* not reported. Fatty acid components which were detected less than 1% in all 5 strains are not shown except C17:1ω6c, which was described in the main text)

Based on comparative phylogenetic analysis using 16S rRNA gene sequences, strain KK2020170^T^ was suggested to belong to the genus *Flavobacterium*. Subsequent whole-genome shotgun sequencing showed that strain KK2020170^T^, *F. haoranii* LQY-7^ T^, and *F. sediminis* MEBiC07310^T^ are closely related but clearly distinct from each other at the species level. Physiological and chemotaxonomic characterization revealed that strain KK2020170^T^ had typical properties as a member of the genus *Flavobacterium.* For example, strain KK2020170^T^ contains MK-6 as the predominant respiratory quinone, forms yellow colonies, shows similar growth phenotypes (i.e., ranges of pH, salt and temperature for growth), and possesses a similar major fatty acid composition as *F. haoranii* LQY-7^ T^ and *F. sediminis* MEBiC07310^T^. However, some characteristics of strain KK2020170^T^ clearly differed from those of its closest neighbor *F. haoranii* LQY-7^ T^, such as the ability to hydrolyze casein; inability to hydrolyze aesculin, gelatin, and starch; minor fatty acid composition; and some biochemical characteristics. We, therefore, conclude that strain KK2020170^T^ represents a distinct species within the genus *Flavobacterium*, for which the name *Flavobacterium okayamense* sp. nov. is proposed.

### Description of *Flavobacterium okayamense* sp. nov.

*Flavobacterium okayamense* (o.ka.ya.men’se. N.L. neut. adj. *okayamense* referring to Okayama in Japan, where the type of strain was isolated).

Cells are Gram-stain-negative, facultatively anaerobic, non-motile, and rod-shaped (0.5–0.6 × 1.0–9.5 μm). Flexirubin-type pigment adsorption by a colony is not detected. Colonies on TSA (after cultivation for 72 h at 30 °C) are yellow, circular, and smooth with low convex. Optimal growth occurs at 30 °C (range 15–40 °C), at pH 7.0 (range, pH 6.5–8.5), and in the presence of 1% (w/v) NaCl (range 1–4%). Cells hydrolyze casein but not starch and tyrosine. Growth occurs on Marine Broth 2216 agar and nutrient agar but not on MacConkey agar. Oxidase and catalase activity is present. In API 20NE tests, hydrolysis of aesculin and gelatin; reduction of nitrate to nitrite; glucose fermentation; indole production; arginine dihydrolase, *β*-galactosidase, and urease activities; and assimilation of arabinose, mannose, maltose, potassium gluconate, *N*-acetylglucosamine, glucose, arabinose, mannitol, caprate, adipate, malate, citrate, and phenyl acetate are negative. In the API ZYM system, alkaline phosphatase, esterase (C4), esterase lipase (C8), leucine arylamidase, valine arylamidase, cystine arylamidase, trypsin, α-chymotrypsin, acid phosphatase, and naphthol-AS-BI-phosphohydrolase activities are present, but other enzyme activities are absent. The major cellular fatty acids are iso-C15:0, iso-C15:1 G, and iso-C17:0 3-OH. The most abundant isoprenoid quinone is MK-6. The major polar lipid is phosphatidylethanolamine.

The type strain, KK2020170^T^ (= ATCC TSD-280^ T^ = NBRC 115344^ T^), was isolated from surface seawater in Kojima Bay (34° 60′ N 133° 99′ E), Okayama, Japan. The genomic DNA G + C content of the type strain is 31.1 mol%.

### Supplementary Information

Below is the link to the electronic supplementary material.

Supplementary file1 (DOCX 664 KB)

## Data Availability

Genomic sequence data of strain KK2020170^T^ is available on the DNA Data Bank of Japan (DDBJ) website (https://ddbj.nig.ac.jp/searchDDBJ) with the accession number AP024749. Raw sequence data of strain KK2020170^T^ is available on the DDBJ Sequence Read Archive (DRA) (https://ddbj.nig.ac.jp/search) under the Biosample accession number PRJDB11590. Strain KK2020170^T^ was deposited to the American Type Culture Collection (ATCC) and the Biological Resource Center, NITE (NBRC) as ATCC TSD-280^T^ and NBRC 115344^T^, respectively.

## References

[CR1] Bae SS, Kim MR, Jung Y, Yang SH, Kwon KK, Baek K (2018). *Flavobacterium sediminis* sp. nov., a starch-degrading bacterium isolated from tidal flat sediment. Int J Syst Evol Microbiol.

[CR2] Bergey DH, Harrison FC, Breed RS, Hammer BW, Huntoon FM (1923). Genus II. Flavobacterium gen. nov. in bergey’s manual of determinative bacteriology.

[CR3] Bernardet JF, Nakagawa Y, Holmes B (2002). Cytophaga-like bacteria of the international committee on systematics of P. Proposed minimal standards for describing new taxa of the family Flavobacteriaceae and emended description of the family. Int J Syst Evol Microbiol.

[CR4] Bernardet JF, Segers P, Vancanneyt M, Berthe F, Kersters K, Vandamme P (1996). Cutting a Gordian knot: emended classification and description of the genus *Flavobacterium*, emended description of the family Flavobacteriaceae, and proposal of *Flavobacterium hydatis* nom. nov. (basonym, *Cytophaga aquatilis* Strohl and Tait 1978). Int J Syst Bacteriol.

[CR5] Bernardet JF, Bowman JP, Genus I, Staley JT, Brown DR (1923). Bergey’s manual of systematic bacteriology. Krieg NR.

[CR6] Bligh EG, Dyer WJ (1959). A rapid method for total lipid extraction and purification. Can J Biochem Physiol.

[CR7] Chun J, Oren A, Ventosa A, Christensen H, Arahal DR, da Costa MS, Rooney AP, Yi H, Xu XW, De Meyer S, Trujillo ME (2018). Proposed minimal standards for the use of genome data for the taxonomy of prokaryotes. Int J Syst Evol Microbiol.

[CR8] Cowan ST, Steel KJ (1965). Manual for the identification of medical bacteria.

[CR9] Felsenstein J (1981). Evolutionary trees from DNA sequences: a maximum-likelihood approach. J Mol Evol.

[CR10] Fitch WM (1971). Toward defining the course of evolution: minimum change for a specific tree topology. Syst Biol.

[CR11] Fujii D, Nagai F, Watanabe Y, Shirasawa Y (2014). *Flavobacterium longum* sp. nov. and *Flavobacterium urocaniciphilum* sp. nov., isolated from a wastewater treatment plant, and emended descriptions of *Flavobacterium caeni* and *Flavobacterium terrigena*. Int J Syst Evol Microbiol.

[CR12] Hou X, Li S, Mao S, Mu W, Guo B, Wei S, Huang M, Zhao Y, Deng H, Sang F, Chen Z, Liu H, Liu A (2022). *Flavobacterium selenitireducens* sp. nov., isolated from the rhizosphere soil of ancient mulberry. Int J Syst Evol Microbiol.

[CR13] Irgang R, Poblete-Morales M, Avendaño-Herrera R (2023). *Flavobacterium pygoscelis* sp. nov., isolated from a chinstrap penguin chick (*Pygoscelis antarcticus*). Int J Syst Evol Microbiol.

[CR14] Jung YJ, Yang SH, Kwon KK, Bae SS (2017). *Echinicola strongylocentroti* sp. nov., isolated from a sea urchin *Strongylocentrotus intermedius*. Int J Syst Evol Microbiol.

[CR15] Kitahara K, Yasutake Y, Miyazaki K (2012). Mutational robustness of 16S ribosomal RNA, shown by experimental horizontal gene transfer in *Escherichia coli*. Proc Natl. Acad Sci USA.

[CR16] Králová S, Busse HJ, Bezdíček M, Sandoval-Powers M, Nykrýnová M, Staňková E, Krsek D, Sedláček I (2021). *Flavobacterium flabelliforme* sp. nov. and *Flavobacterium geliluteum* sp. nov., two multidrug-resistant psychrotrophic species isolated from antarctica. Front Microbiol.

[CR17] Kumar S, Stecher G, Tamura K (2016). MEGA7: molecular evolutionary genetics analysis version 7.0 for bigger datasets. Mol Biol Evol.

[CR18] Máté R, Kutasi J, Bata-Vidács I, Kosztik J, Kukolya J, Tóth E, Bóka K, Táncsics A, Kovács G, Nagy I, Tóth Á (2022). *Flavobacterium hungaricum* sp. nov. a novel soil inhabitant, cellulolytic bacterium isolated from plow fields. Arch Microbiol.

[CR19] McBride MJ, Rosenberg E, DeLong EF, Lory S (2014). The family flavobacteriacea. The prokaryotes: other major lineages of bacteria and the archaea.

[CR20] Meier-Kolthoff JP, Auch AF, Klenk HP, Göker M (2013). Genome sequence-based species delimitation with confidence intervals and improved distance functions. BMC Bioinform.

[CR21] Minnikin DE, Collins MD, Goodfellow M (1979). Fatty acid and polar lipid composition in the classification of *Cellulomonas*, *Oerskovia*, and related taxa. J Appl Microbiol.

[CR22] Miyazaki K, Wiseschart A, Pootanakit K, Kitahara K (2020). Complete genome sequence of *Vibrio rotiferianus* Strain AM7. Microbiol Resour Announc.

[CR23] Lane JD, Stackebrandt E, Goodfellow M (1991). 16S/23S rRNA sequencing. Nucleic acid techniques in bacterial systematics.

[CR24] Pritchard L, Glover RH, Humphris S, Elphinstone JG, Toth IK (2016). Genomics and taxonomy in diagnostics for food security: soft-rotting enterobacterial plant pathogens. Anal Methods.

[CR100] Saha P, Chakrabarti T (2006). Flavobacterium indicum sp. nov., isolated from warm spring water in Assam, India. Int J Syst Evol Microbiol.

[CR25] Saitou N, Nei M (1987). The neighbor-joining method: a new method for reconstructing phylogenetic trees. Mol Biol Evol.

[CR26] Sasser M (1990). Identification of bacteria by gas chromatography of cellular fatty acids, MIDI Technical Note 101.

[CR27] Seo J, Peng Y, Jiang L, Lee SB, Jeong RD, Park SJ, Kim CY, Choi M, Lee J (2022). *Flavobacterium endoglycinae* sp. nov., an endophytic bacterium isolated from soybean (Glycine max L. cv. Gwangan) stems. Int J Syst Evol Microbiol.

[CR28] Smibert RM, Gerhardt P (1994). Phenotypic characterization. Methods for general and molecular bacteriology.

[CR29] Sun H, Zheng H, Wang X, Jiang Y, Liao B, Li A, Xiao B (2022). *Flavobacterium coralii* sp. nov., a marine bacterium isolated from coral culture seawater. Int J Syst Evol Microbiol.

[CR30] Tamaoka J, Katayama-Fujimura Y, Kuraishi H (1983). Analysis of bacterial menaquinone mixtures by high-performance liquid chromatography. J Appl Microbiol.

[CR31] Watanabe K, Kitamura T, Ogata Y, Shindo C, Suda W (2022). *Flavobacterium ammonificans* sp. nov. and *Flavobacterium ammoniigenes* sp. nov., ammonifying bacteria isolated from surface river water. Int J Syst Evol Microbiol.

[CR32] Yang SH, Seo HS, Lee JH, Kim SJ, Kwon K (2016). *Pseudofulvibacter gastropodicola* sp. nov., isolated from a marine conch and emended descriptions of the genus Pseudofulvibacter Yoon, 2013 and *Pseudofulvibacter geojedonensis*. Int J Syst Evol Microbiol.

[CR33] Yu H, Taniguchi M, Uesaka K, Wiseschart A, Pootanakit K, Nishitani Y, Murakami Y, Ishimori K, Miyazaki K, Kitahara K (2019). Complete genome sequence of *Staphylococcus arlettae* Strain P2, isolated from a laboratory environment. Microbiol Resour Announc.

[CR34] Zhang GQ, Liu Q, Liu HC, Zhou YG, Xin YH (2019). *Flavobacterium ranwuense* sp. nov., isolated from glacier. Int J Syst Evol Microbiol.

[CR35] Zhang J, Jiang RB, Zhang XX, Hang BJ, He J, Li SP (2010). *Flavobacterium haoranii* sp. nov., a cypermethrin-degrading bacterium isolated from a wastewater treatment system. Int J Syst Evol Microbiol.

