# Supplementary material for: Flavobacterium okayamense sp. nov. isolated from surface seawater
Source: Arch Microbiol. 2023 Sep 29;205(10):346. doi: 10.1007/s00203-023-03682-x (PMC10542280; doi:10.1007/s00203-023-03682-x)
Supplement: Supplementary file 1 — Supplementary file1 (DOCX 664 KB) [file 203_2023_3682_MOESM1_ESM.docx]

**Table S1.** Genome information of *F. okayamense* strain KK2020170^T^ according to the proposed minimal standards of genome data for prokaryotic taxonomy

|  | ***Flavobacterium okayamense* strain KK2020170^T^**  (=ATCC TSD-280^T^=NBRC 115344^T^) |
| --- | --- |
| DDBJ BioProject  DDBJ Biosample | PRJDB11590  PRJDB11590 |
| DDBJ Accession numbers. | AP024749 |
| Sequencing technology | MiSeq (Illumina) and GridION (Oxford Nanopore Technology) |
| Assembly method | De novo assembly (Unicycler v.0.4.8) |
| Assembly name | *Flavobacterium* sp. KK2020170 DNA, complete genome |
| Genome coverage | 126.1 |
| Annotation pipeline | Prokaryotic Genome Annotation Pipeline |
| Annotation method | DFAST v.1.2.4 (https://dfast.nig.ac.jp) |
| Genome size (bp) | 2,781,077 |
| DNA G+C content (mol%) | 31.1 |
| Total genes | 2574 |
| Protein-coding genes | 2574 |
| rRNA genes | 9 |
| tRNA genes | 51 |


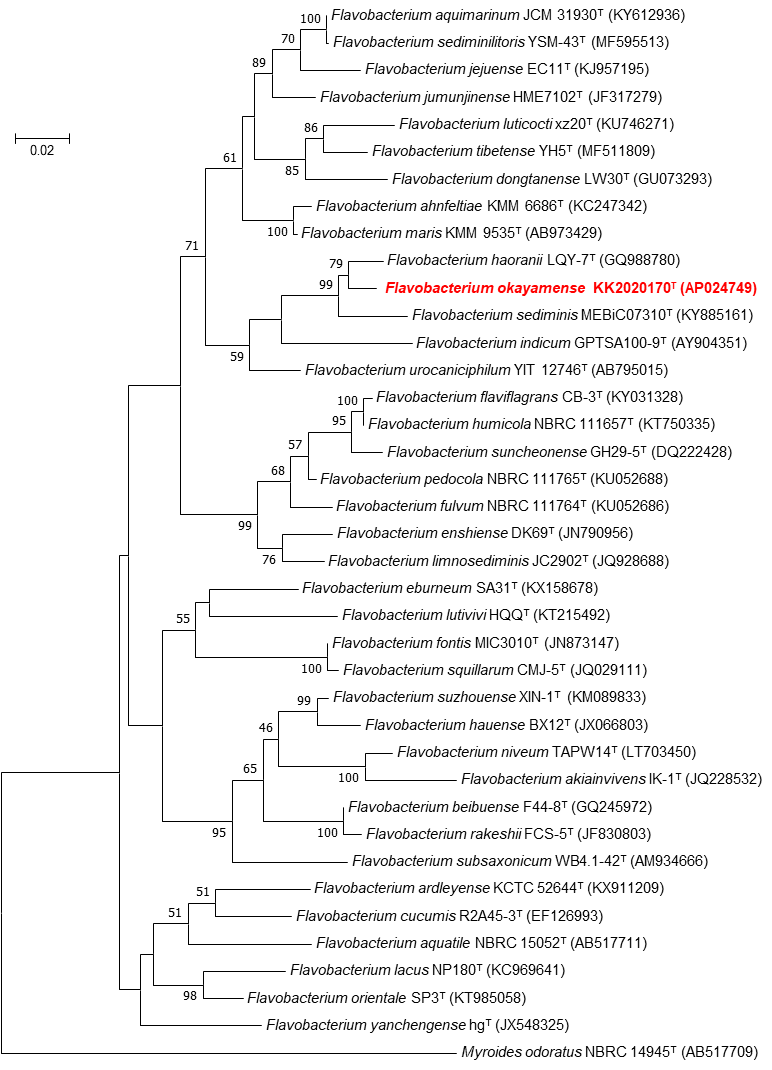


**Fig. S1.** A maximum-likelihood tree was built on a nearly complete 16S rRNA gene sequence (1439 unambiguously aligned bp) demonstrating the connection between *F. okayamense* strain KK2020170^T^ and 37 adjacent type strains in the genus *Flavobacterium*. At branch points, Bootstrap values (given as percentages of 1000 replicates) with >50% values are displayed. *Flavobacterium aquatile* is the type species in the genus *Flavobacterium*. *Myroides odoratus* NBRC 14945^T^ (AB517709) was employed an outgroup. Bar, 0.01 substitutions per nucleotide position.

**A**


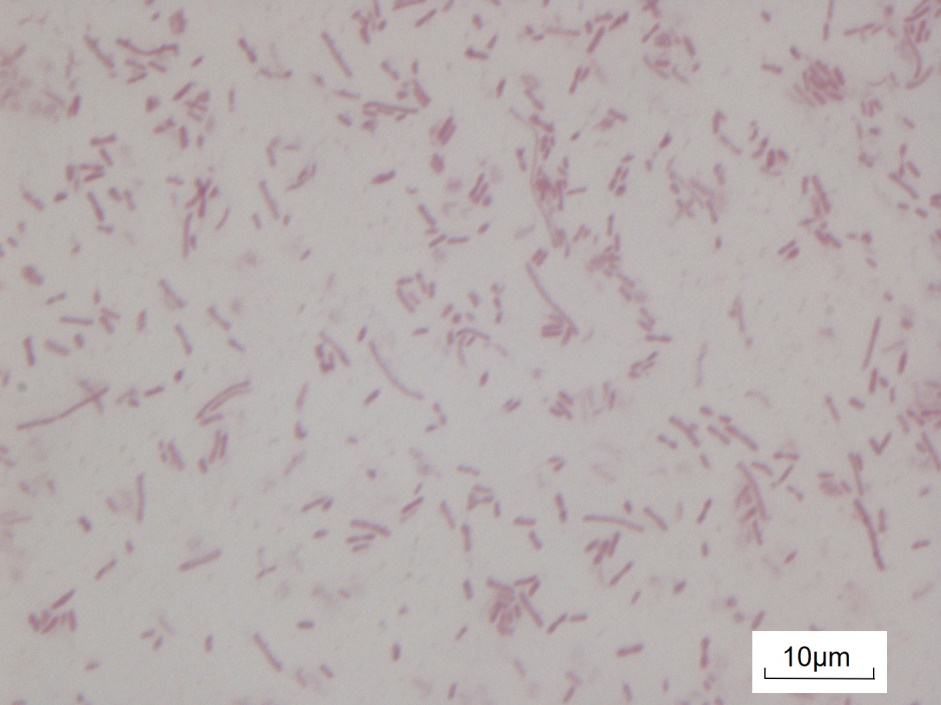


**B**


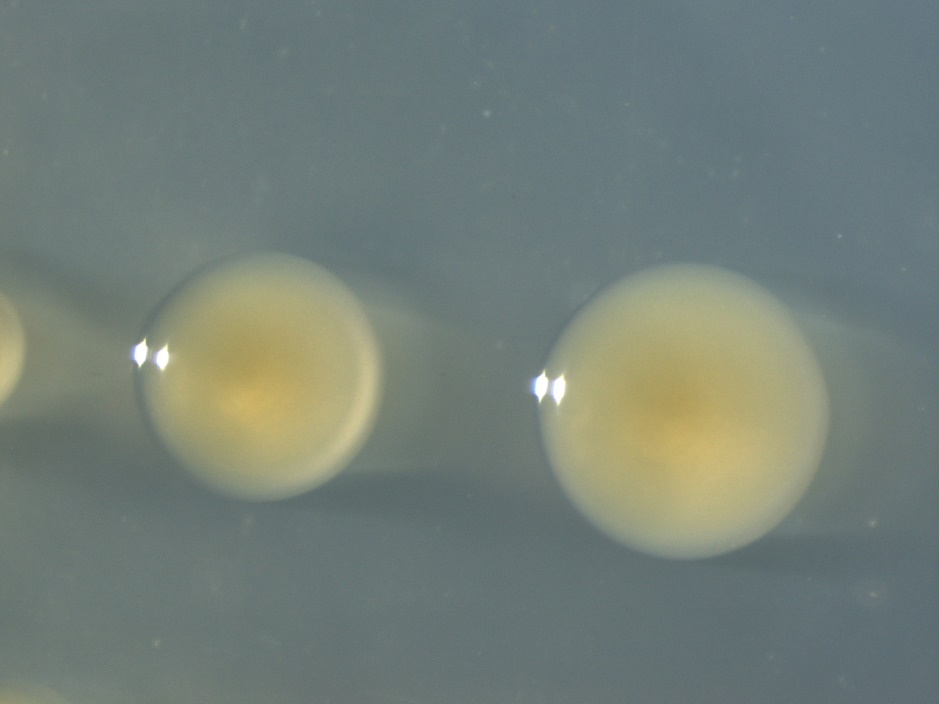


**Fig. S2.** Colony and cell morphologies of *F. okayamense* strain KK2020170^T^. **A,** Microscopic image of strain KK2020170^T^ cells. Cells were Gram-stained and visualized under a phase-contrast microscope at ×1000 magnification. Bar, 10 μm. **B,** A photograph of colonies grown on a TSA plate. Typical colony diameter was from 1.3 to 2.0 mm after 72h incubation at 30°C.

**Fig. S3.** Polar lipid profiles of *F. okayamense* strain KK2020170^T^ determined by 2D thin-layer chromatography. PE, phosphatidylethanolamine; LPE, lyso-phosphatidyl-ethanolamine, AL1-3, aminolipids 1–3; UL1–5; unidentified lipids. Chloroform, methanol, and water (65:25:4 [v/v]) were used in the first dimension, and chloroform, acetic acid, and methanol/water (80:18:12:5 [v/v]) were used in the second dimension. The total lipid components were detected using 5% (w/v) molybdophosphoric acid in isopropanol.
